# Identification of mitophagy‐associated proteins profile as potential plasma biomarkers of idiopathic Parkinson's disease

**DOI:** 10.1111/cns.14532

**Published:** 2023-11-21

**Authors:** Shuangjie Qian, Haijun He, Xi Xiong, Ruixue Ai, Wenwen Wang, Huimin Zhu, Qianqian Ye, Shuoting Zhou, Hilde Nilsen, Chenglong Xie

**Affiliations:** ^1^ Department of Neurology The First Affiliated Hospital of Wenzhou Medical University Wenzhou China; ^2^ Department of Clinical Molecular Biology University of Oslo and Akershus University Hospital Lørenskog Norway; ^3^ The Center of Traditional Chinese Medicine The Second Affiliated Hospital and Yuying Children's Hospital of Wenzhou Medical University Wenzhou China; ^4^ Department of Microbiology Oslo University Hospital Oslo Norway; ^5^ Institute of Clinical Medicine, Department of Clinical Molecular Biology University of Oslo Oslo Norway; ^6^ Unit of Precision Medicine Akershus University Hospital Nordbyhagen Norway; ^7^ Key Laboratory of Alzheimer's Disease of Zhejiang Province Institute Of Aging, Wenzhou Medical University Wenzhou Zhejiang China; ^8^ Oujiang Laboratory Wenzhou Zhejiang China; ^9^ Department of Geriatrics, Geriatric Medical Center The First Affiliated Hospital of Wenzhou Medical University Wenzhou Zhejiang China

**Keywords:** biomarkers, diagnosis, MAPs, mitophagy‐associated proteins, Parkinson's disease

## Abstract

**Background:**

Despite extensive work to identify diagnostic plasma markers for Parkinson's disease (PD), there are still no accepted and validated surrogate biomarkers. Mitophagy‐associated proteins (MAPs), including PTEN‐induced putative kinase 1 (PINK1), Parkin, phosphoglycerate mutase 5 (PGAM5), BCL2 interacting protein 3 (BNIP3), and phosphorylated‐TBK1 (p‐TBK1), are, to our best knowledge, not well studied as a panel of biomarkers of neurodegeneration in PD.

**Methods:**

The study population comprised 116 age‐matched controls (HC), 179 PD patients, alongside and 90 PD syndromes (PDs) divided between two cohorts: (i) the modeling cohort (cohort 1), including 150 PD, 97 HC, and 80 PDs; and (ii) the validated cohort (cohort 2), including 29 PD, 19 HC, and 10 PDs.

**Results:**

MAPs are elevated in the plasma of PD patients. PINK1, Parkin, and PGAM5 displayed the top three measurable increase trends in amplitude compared to BNIP3 and p‐TBK1. Moreover, the area under the curve (AUC) values of PINK1, PGAM5, and Parkin were ranked the top three MAP candidates in diagnosis accuracy for PD from HC, but the MAPs make it hard to differentiate PD from PDs. In addition, there are higher plasma PINK1‐Parkin levels and prominent diagnostic accuracy in A‐synuclein (+) subjects than in A‐synuclein (−) subjects.

**Conclusions:**

These results uncover that plasma MAPs (PINK1, Parkin, and PGAM5) may be potentially useful diagnostic biomarkers for PD diagnosis. Studies on larger cohorts would be required to test whether elevated plasma MAP levels are related to PD risk or prognosis.

## Graphical abstract

Subjects with Parkinson's disease (PD) or PDs exhibit higher mitophagy‐associated protein (MAP) levels compared to the CN group, but the differences in values between PD and PDs did not reach significance. The AUCs of PINK1, PGAM5, and Parkin were ranked as the top three MAP candidates in diagnosis accuracy for PD from CN. Higher plasma PINK1‐Parkin levels and prominent diagnostic accuracy in A‐syn (+) subjects are higher than in A‐syn (−) subjects.

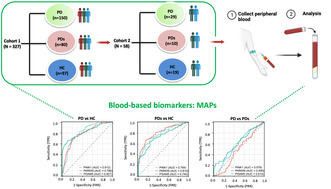

## 1 INTRODUCTION

Parkinson's disease (PD) is the most prevalent devastating neurodegenerative movement disorder, suffering roughly 1% of the population aged 65 or older worldwide, for which there is no causative treatment.^1^ The clinical motor symptoms of PD result from the insufficiency of dopaminergic neurons in the Substantia Nigra pars compacta (SNpc) in the midbrain, simultaneously occurring in their axon terminals, which project the dorsal striatum.^2^ At present, the diagnosis of PD is mainly based on an individuals' history, physical examination, and response to dopaminergic medication. History can include diverse prodromal features, characteristic movement disturbances (such as tremors, bradykinesia, and rigidity), and psychological as well as cognitive problems et al.^3^ Moreover, the clinical diagnosis can be elevated through imaging tools that are advantageous for detecting neurodegenerative damage. Lewy bodies are the pathologic hallmark of PD, which is a neuronal inclusion largely made up of α‐synuclein misfolding and aggregations.^4^ However, even when the aforementioned criteria are correctly used, the risk of misdiagnosis is still significant because of numerous clinical overlaps among Parkinsonian disorders.^5^ Although positron emission tomography (PET) and single‐photon emission computerized tomography (SPECT) techniques are very sensitive, they are expensive, not specific for PD, and involve radiation exposure.^6^ Thus, exploring new mechanisms and corresponding feasible biomarkers for the early identification of patients with PD is urgently needed. Compared to CSF, plasma‐based biomarkers are under investigation because they would provide a non‐invasive method.

Of interest, damaged mitochondria and their associated oxidative stress, adenosine triphosphate (ATP) deletion, reactive oxygen species (ROS) production, and neuroinflammation are common characteristics in PD subject brain samples and seen in animal models of PD.^7^, ^8^ Biochemical and genetic research has identified two genes commonly mutated in familial, autosomal recessive PD, namely *PINK1* and *Parkin*, which function in the same pathway to modulate mitophagy and mitochondrial quality control.^9^ Mitophagy is a form of selective autophagy dedicated to eliminating damaged mitochondria to maintain cellular integrity and homeostasis and is beneficial for neuroprotection.^10^ PINK1 localizes on the outer membrane of damaged mitochondria, and its kinase activity is required for Parkin translocation to the mitochondria, where Parkin ubiquitinates outer membrane proteins (such as FUNDC1, BNIP3, FKBP8, etc.) to trigger selective autophagy.^11^ Historically, Nicholas et al. reported that hereditary early‐onset PD was caused by mutations in *PINK1* as the core factor to initiating the mitophagy process. They identified two homozygous mutations influencing the PINK1 kinase domain from three consanguineous PARK6‐carrier PD families.^12^ In vitro, mitochondrial dysfunction in *PINK1* ^*−/−*^ human dopaminergic neurons and lowered autophagic flux can be rescued by parkin expression, indicating PINK1‐parkin‐dependent mitophagy is involved in PD pathogenesis.^13^

Notably, despite many efforts to screen biochemical plasma markers for PD diagnosis, there are still no accepted and validated surrogate biomarkers. Mitophagy‐associated proteins (MAPs), including PINK1, Parkin, PGAM5, BNIP3, and phosphorylated‐TBK1 (p‐TBK1), are, to our best knowledge, not well studied as a panel of biomarkers of neurodegeneration in PD. The primary aim of this study was to decipher the potential role of the mitophagy pathway proteins in idiopathic PD subjects and investigate whether the diagnosis is related to MAP levels and whether the levels predict motor and cognitive progression. Moreover, considering that mitophagy was identified as a pathogenic mechanism in animal models of PD, we hypothesized that MAPs are dysregulated due to impaired mitochondrial function.

## 2 METHODS

### 2.1 Standard protocol approvals, registrations, and patients consent

All patients in this study were consecutively recruited from the First Affiliated Hospital of Wenzhou Medical University. The study was approved by the institutional Ethics Board Committee of the Wenzhou Medical University First Affiliated Hospital. All participants provided their written informed consent prior to joining this study.

### 2.2 Study population

**FIGURE 1:**
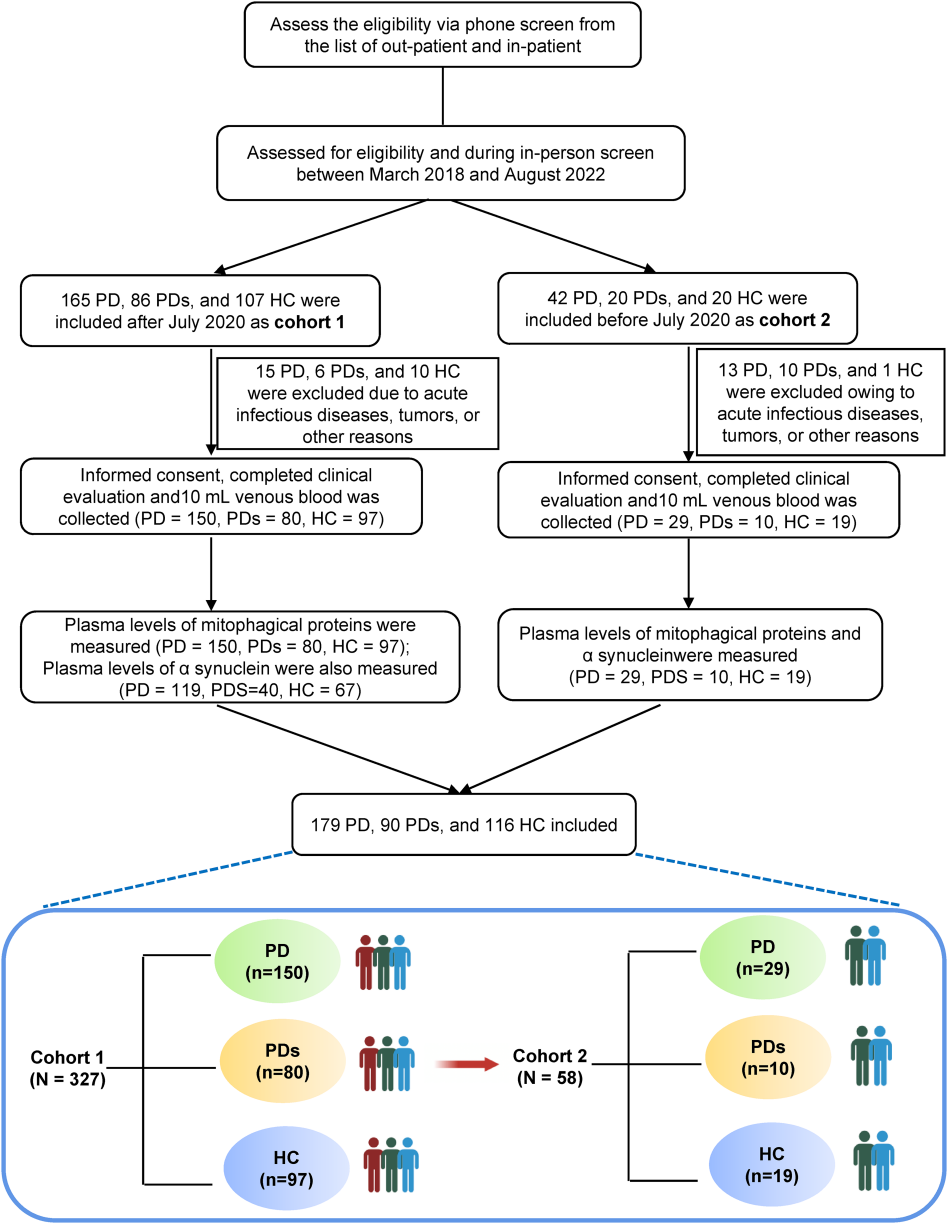
Flow chart of the study design and selection process.

See Figure 1 for a flowchart of this study's selection process. All participants were recruited from the First Affiliated Hospital of Wenzhou Medical University between October 2018 and August 2022 and were divided into three groups: HC, PD, and PD syndromes (PDs). Patients with PD were classified as having clinically established or probable PD based on the Movement Disorder Society Clinical Diagnostic Criteria.^14^ Participants in the PDs group were defined as patients with Parkinsonian syndrome, including MSA (multiple system atrophy), PSP (progressive supranuclear palsy), DLB (dementia with Lewy bodies), or vascular PD. Controls comprised outpatients or inpatients who were neurologically unaffected participants without a history of Parkinsonism, psychiatric disorders, brain trauma or stroke, or cancer, etc. The participants recruited after March 2020 were labeled as Cohort 1, while the rest were labeled as Cohort 2. They were recruited at the same center but adopted different kits.

### 2.3 Diagnostic criteria for PD, PDs, and HC

All the individuals included in the study were clinically assessed using standard scales that evaluated neuropsychiatric, cognitive, and movement disorder components. PD patients were diagnosed according to the Movement Disorder Society (MDS) clinical diagnostic criteria.^14^ PDs comprised MSA, PSP, DLB, or VPD. In all subjects, those who had other neurodegenerative diseases, such as Alzheimer's disease (AD), Wilson's disease (WD), acute infectious diseases, tumors, etc., were excluded. HC participants were spouses or friends of patients with PD or PDs and were in neurologically normal condition. Clinical PD and PDs diagnoses were all performed by experienced movement disorders specialty‐trained neurologists.

### 2.4 Neuropsychological evaluation

Participants were analyzed using the Unified Parkinson's Disease Rating Scale (UPDRS)^15^ and Hoehn–Yahr staging^16^ to evaluate the motor symptoms and progression stage of PD. The Chinese version of the Mini‐Mental State Examination (MMSE) was used for cognitive examination, with adjustment of the cutoff score for cognitive impairment according to the years of education as follows: illiterate, ≤17 points; primary school education, ≤20 points; and postsecondary education or above, ≤24 points.^17^, ^18^ An examination of emotional aspects was performed using the Hamilton Depression Rating Scale (HAMD) and the Hamilton Anxiety Rating Scale (HAMA). Specifically, depression and anxiety were defined as HAMD score ≥17 points and HAMA score ≥14 points, respectively. The REM sleep behavior disorder questionnaire‐Hong Kong (RBDQ‐HK) was used to detect REM sleep behavior disorder (RBD), with a cutoff value of ≥18 points.^19^ All the examinations were done in the “on” state of the disease.

### 2.5 Measurement of plasma biomarkers

Plasma samples were obtained through blood centrifugation (3000 × g for 10 min) and stored at −80°C until subsequent analysis. We used the EDTA anticoagulant tube to collect blood and delivered it to centrifugation within 1 h. Plasma PNK1, Parkin, PGAM5, BNIP3, p‐TBK1, total a‐syn, phosphorylated a‐syn (p‐asyn), and a‐syn oligomer levels were determined using enzyme‐linked immunosorbent assay (Jianglai Biotechnology Company, Shanghai, China, http://www.jonln.com). The detailed information on these kits is as follows: No: JL11175 (PINK1), JL11195 (Parkin), JL11208 (PGAM5), JL34153 (BNIP3), JL11283 (p‐TBK1), JL12231 (total a‐syn), JL12598 (p‐asyn), and JL41188 (a‐syn oligomer), respectively. The samples were processed by a laboratory technician blinded to all clinical data, following the manufacturer's instructions. Briefly, 80 μL of standard solution and 20 μL of samples (5 × diluted) were pipetted into the wells of 96‐well plates. Next, 100 μL of antibody‐horse radish peroxidase conjugate (MyBioSource, United States) was added to standard and sample wells, which were covered using an adhesive strip and incubated for 60 min at 37°C. After washing four times, the plates were incubated in a tetramethylbenzidine substrate for 15 min at 37°C. Additionally, the reactions were stopped with H_2_SO_4_. The plates were read at the 450‐nm wavelength. All samples were run in triplicate.

### 2.6 Statistical analyses

Continuous variables were assessed for normality using the Kolmogorov–Smirnov test, histogram, and Q–Q plot. Variables were expressed as the mean (SD) or median [IQR] and assessed by analysis of covariance (ANCOVA) or Kruskal–Wallis test, followed by Bonferroni‐corrected post hoc comparisons. Categorical variables were presented as numbers (percentages) and were compared using the Chi‐square test followed by Bonferroni correction. The associations between plasma biomarkers and neuropsychological evaluation scales were tested using Pearson correlations and restricted cubic spline (RCS).^20^ RCS was fitted between biomarkers and neuropsychological scores in the PD group adjusted for age, sex, and education, with three knots fixed at the 10th, 50th, and 90th percentiles, and *p*‐values for nonlinearity were calculated using the Wald chi‐square test. Random forest classifier^21^ was performed with all the plasma biomarkers, neuropsychological scores, and demographic factors ranked according to the proportion of importance (the number of decision trees was 100). Partial participants (HC = 67, PD = 119, PDs = 40) were analyzed for the model of Random Forest and followed combined models in cohort 1 due to the lack of plasma a‐syn examination, while the lack of data on plasma BNIP3 and p‐TBK1 was added by multiple imputations. Then, optimal model indices were further screened using logistic regression analysis and Akaike information criterion (AIC).^22^ Finally, the area under the curve (AUC) in receiver operating characteristic (ROC) curves was used to evaluate the predictive utility of plasma biomarkers alone or in combined models, and the difference in the AUC was determined using DeLong statistics. All the basic analyses were practiced in cohort 1, and the combined models were also validated in cohort 2 to see the stability of the constructed models. Statistical analyses were performed using R version 4.1 and Python version 3.9. Statistical significance was set at a two‐tailed *p* < 0.05.

## 3 RESULTS

### 3.1 Baseline phenotypic characteristics of the cohorts

For the modeling cohort, a total of 385 participants were selected, comprising 116 individuals with HC, 179 clinically diagnosed with PD, and 90 individuals subsequently characterized as having PDs. The basic information includes average age, sex ratio, height, weight, BMI, education, disease duration, UPDRS, Hoehn–Yahr stage, HAMA, HAMD, RBD, ADL, and the levels of a‐synuclein as well as MAPs provided. Patients with PDs were older at the time of recruitment and sampling of biomaterials. There was a high male/female ratio with PD and PDs (56% of PD and 58.1% of PDs) compared to HC (43.3%) and mild low education levels in the PD group, but no significance (Table 1 shows demographic data for each group investigated here). To explore a possible correlation between increased expression of MAPs and PD in this cohort, we quantified our cohort's MAP levels in plasma. Moreover, PDs were characterized by more serious motor symptoms and psychological assessment than PD subjects based on these assessment scales. In parallel, a similar pattern of basic participant characteristics was observed in a small‐size subset validated cohort (*N* = 58), also described in Table 1. The participants in these two cohorts are recruited from the same center, but the samples are independent.

**TABLE 1:** Baseline characteristics for the modeling and validated cohorts.

Characteristics	Modeling cohort						Validated cohort					
	HC (*N* = 97)	PD (*N* = 150)	PDs (*N* = 80)	PD vs. HC^a^	PDs vs. HC^a^	PD vs. PDs^a^	HC (*N* = 19)	PD (*N* = 29)	PDs (*N* = 10)	PD vs. HC^a^	PDs vs. HC^a^	PD vs. PDs^a^
Age (years)	64.0 [59.0; 69.0]	66.5 [60.2; 72.0]	67.0 [62.0; 74.5]	0.036	0.022	0.301	68.0 [61.0; 74.0]	63.0 [62.0; 69.0]	75.0 [68.2; 78.0]	0.375	0.098	0.027
Female (%)^b^	55 (56.7%)	66 (44.0%)	31 (41.9%)	0.116	0.116	0.876	13 (68.4%)	17 (58.6%)	4 (40.0%)	0.703	0.697	0.697
Height (cm)	162 (7.05)	161 (8.28)	161 (8.50)	0.329	0.551	0.983	159 (7.03)	160 (8.61)	160 (6.40)	0.849	0.912	1.000
Weight (kg)	63.1 (9.34)	61.8 (10.6)	62.7 (9.93)	0.561	0.963	0.788	62.7 (8.17)	63.1 (17.5)	62.8 (8.75)	0.992	1.000	0.998
BMI	24.0 (2.94)	23.9 (3.15)	24.3 (4.04)	0.974	0.770	0.609	24.7 (2.54)	24.3 (5.02)	24.6 (4.31)	0.947	0.997	0.985
Education (years)	5.00 [0.00; 8.00]	4.00 [0.00; 6.38]	3.00 [0.00; 6.00]	0.773	0.773	0.773	2.00 [0.00; 4.50]	3.00 [0.00; 8.00]	2.50 [0.00; 4.00]	0.576	0.962	0.576
Disease History (years)	–	3.50 [1.62; 7.00]	2.00 [1.00; 4.00]	–	–	0.001	–	4.00 [2.00; 5.00]	2.75 [1.25; 6.50]	–	–	0.697
UPDRS	–	41.5 [27.2; 53.0]	41.0 [28.0; 60.0]	–	–	0.457	–	36.0 [28.0; 52.0]	62.5 [56.5; 74.2]	–	–	0.012
I	–	2.00 [1.00; 4.00]	2.00 [1.00; 4.00]	–	–	0.236	–	2.00 [1.00; 3.00]	3.50 [2.00; 6.25]	–	–	0.099
II	–	11.0 [7.00; 16.0]	13.0 [8.00; 17.0]	–	–	0.386	–	11.0 [10.0; 14.0]	17.5 [13.2; 20.0]	–	–	0.061
III	–	24.5 [15.2; 34.0]	25.0 [17.0; 36.0]	–	–	0.661	–	23.0 [14.0; 33.0]	39.5 [27.8; 46.8]	–	–	0.007
IV	–	2.00 [0.00; 3.00]	1.00 [0.00; 2.00]	–	–	0.038	–	3.00 [0.00; 5.00]	3.50 [1.25; 4.75]	–	–	0.625
H–Y stage	–	2.50 [1.50; 3.00]	3.00 [2.00; 4.00]	–	–	0.049	–	2.00 [1.50; 2.50]	3.00 [3.00; 4.00]	–	–	0.020
MMSE	24.0 [21.0; 27.0]	23.0 [18.0; 27.0]	18.0 [12.0; 23.2]	0.039	<0.001	<0.001	24.0 [20.0; 27.0]	26.0 [20.0; 28.0]	17.5 [11.5; 20.0]	0.899	0.061	0.061
HAMD	3.00 [0.00; 5.00]	5.00 [2.00; 9.00]	5.00 [3.00; 9.00]	<0.001	<0.001	0.916	2.00 [0.00; 5.50]	6.00 [3.00; 9.00]	7.00 [3.50; 8.50]	0.022	0.034	0.884
HAMA	3.00 [1.00; 7.00]	7.00 [4.00; 13.0]	7.00 [4.00; 11.0]	<0.001	<0.001	0.367	3.00 [0.00; 9.00]	14.0 [8.00; 17.0]	7.00 [5.25; 8.75]	<0.001	0.152	0.036
RBDQ‐HK	3.00 [1.00; 8.00]	13.0 [3.00; 31.0]	4.00 [1.00; 19.0]	<0.001	0.044	0.001	5.00 [3.50; 7.50]	24.0 [8.00; 46.0]	12.5 [3.75; 38.2]	0.003	0.273	0.520
ADL	20.0 [20.0; 20.0]	26.0 [21.0; 34.8]	35.0 [23.0; 46.5]	<0.001	<0.001	<0.001	20.0 [20.0; 20.0]	22.0 [20.0; 26.0]	50.5 [31.2; 58.8]	<0.001	<0.001	0.001

*Note*: Continuous variables were assessed for normality by the Kolmogorov–Smirnov test, P–P plot and Q–Q plot. Data are expressed as mean (SD) (normal distribution), median [IQR] (abnormal distribution) or *n* (%) (categorical variable); *p*‐values of continuous variables obtained from One‐Way ANOVA (normal distribution) or Kruskal–Wallis test (abnormal distribution) followed by Bonferroni corrected post hoc comparisons, and UPDRS and H–Y stage were only compared between PD and PDs by Student's *t*‐test; *p*‐values of categorical variables obtained from chi‐squared test corrected by Bonferroni.

Abbreviations: ADL, Activity of Daily Living Scale; BMI, body mass index; HAMD, Hamilton Depression Scale; HAMA, Hamilton Anxiety Scale; HC, healthy control; MMSE, Mini‐Mental State Examination; PD, Parkinson disease; PDs, Parkinsonian syndrome; RBDQ‐HK, REM sleep behavior disorder questionnaire‐Hong Kong; UPDRS, unified Parkinson's disease rating scale.

^a^ *p*‐values of two groups.

^b^ Number of individuals and percent within group.

### 3.2 MAPs are elevated in the plasma of PD patients

**FIGURE 2:**
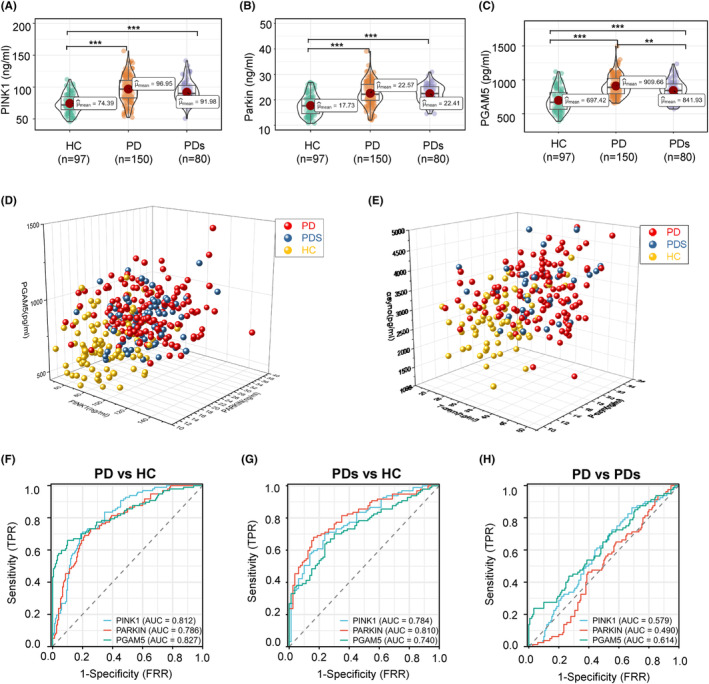
Plasma‐derived mitophagy‐associated proteins (MAPs) and a‐synuclein‐related proteins (ASPs) concentrations and diagnostic accuracy across clinically defined diagnostic profiles. (A–C) Distribution of MAPs (PINK1, Parkin, and PGAM5) concentrations across clinically defined diagnostic groups; The distribution dots of MASPs (D) and ASPs (E) in three‐dimensional space across three groups. The diagnosis accuracy of MAPs (I–K) in the context of Parkinson disease (PD) versus healthy control (HC) (F), PDs versus HC (G), and PD versus PDs (H). *p < 0.05, **p < 0.01, ***p < 0.001 compared to the healthy control group.

As a first step, we tested the distribution of plasma‐derived MAP levels (PINK1, Parkin, PGAM5, BNIP3, and p‐TBK1) in the modeling cohort. We hypothesized that mitochondrial dysfunction in patients with PD would be associated with higher MAP levels compared to healthy control, as a reflection of ongoing mitophagy. Consistent with this hypothesis, plasma PINK1, Parkin, and PGAM5 concentrations were significantly higher in PD (97.0 ± 20.5 ng/mL, 22.6 ± 4.67 ng/mL, 910 ± 147 pg/mL, respectively) and PDs (92.0 ± 17.0 ng/mL, 22.4 ± 3.45 ng/mL, 842 ± 150 pg/mL, respectively) as compared to the HC group (74.4 ± 14.7 ng/mL, 17.7 ± 3.96 ng/mL, 697 ± 167 pg/mL, respectively) (Table 2 and Figure 2A–C). Oppositely, the levels of p‐TBK1 (HC: 9.59 ± 1.91 ng/mL, PD: 10.5 ± 2.10 ng/mL, PDs: 9.73 ± 1.70 pg/mL, respectively) and BNIP3 (HC: 13.9 ± 3.32 ng/mL, PD: 15.7 ± 3.56 ng/mL, PDs: 17.1 ± 3.23 pg/mL, respectively) were similar among the groups (Table 2 and Figure S1A,B). There was no difference between PD and PDs, where all MAPs were analyzed using pairwise analysis (*p* > 0.05). PINK1, Parkin, and PGAM5 displayed the top three measurable increase trends in amplitude compared to BNIP3 and p‐TBK1. PINK1 localizes on the mitochondrial outer membrane and induces Parkin and a series of membrane proteins to trigger selective autophagy. Therefore, it is plausible that elevated levels are seen for both of these MAPs. As expected, PD or PDs subjects exhibited higher total a‐synuclein (35.9 ± 6.26 ng/mL, 35.2 ± 5.95 ng/mL, respectively), phosphorylated a‐synuclein (18.4 ± 3.13, 18.4 ± 2.75 ng/mL), and a‐synuclein oligomer (3394 ± 663, 3445 ± 625 pg/mL) levels than the HC group (Table 2 and Figure S1C–E), but the differences in values between PD and PDs did not reach significance. Thus, MAPs and a‐synuclein‐related proteins (ASPs) are unsuitable as biomarkers to differentiate PD from PDs. The distribution dots in Figure 2D,E demonstrated the high value of MAPs and ASPs located in PD and PDs regions. Significantly, Table 2 indicates the levels of all proteins measured in the modeling cohort and validated cohort are mildly different, maybe due to the kits used in the second cohort from the other Biotech company (www.mmbio.cn).

**TABLE 2:** Plasma‐derived multiple biomarker levels of these two cohorts.

Biomarkers	Modeling cohort^a^						Validated cohort^c^					
	HC (*N* = 97)	PD (*N* = 150)	PDs (*N* = 80)	PD vs. HC	PDs vs. HC	PD vs. PDs	HC (*N* = 19)	PD (*N* = 27)	PDs (*N* = 10)	PD vs. HC	PDs vs. HC	PD vs. PDs
PINK1 (ng/mL)	74.4 (14.7)	97.0 (20.5)	92.0 (17.0)	<0.001	<0.001	0.117	66.6 (4.62)	74.5 (6.72)	71.8 (6.10)	<0.001	0.076	0.438
Parkin (ng/mL)	17.7 (3.96)	22.6 (4.67)	22.4 (3.45)	<0.001	<0.001	0.956	17.1 (1.75)	18.6 (2.63)	17.7 (1.76)	0.068	0.767	0.524
PGAM5 (pg/mL)	697 (167)	910 (147)	842 (150)	<0.001	<0.001	0.005	778 (63.7)	828 (63.1)	789 (89.5)	0.040	0.911	0.264
P‐TBK1 (ng/mL)^a^	9.59 (1.91)	10.5 (2.10)	9.73 (1.70)	0.056	0.946	0.064	9.24 (0.79)	9.55 (0.83)	9.56 (1.23)	0.467	0.620	0.999
BNIP3 (ng/mL)^a^	13.9 (3.32)	15.7 (3.56)	17.1 (3.23)	0.027	<0.001	0.068	13.7 (0.74)	13.5 (1.06)	13.4 (1.11)	0.656	0.723	0.993
T‐αsyn (ng/mL)^b^ ^,^ ^c^	28.9 (5.61)	35.9 (6.26)	35.2 (5.95)	<0.001	<0.001	0.804	–	–	–	–	–	–
P‐αsyn (ng/mL)^b^	15.8 (2.60)	18.4 (3.13)	18.4 (2.75)	<0.001	<0.001	1.000	15.3 (1.26)	16.4 (1.49)	16.3 (1.38)	0.027	0.202	0.953
Αsy‐no (pg/mL)^b^	2647 (658)	3394 (663)	3445 (625)	<0.001	<0.001	0.903	2406 (209)	2494 (236)	2574 (209)	0.381	0.141	0.595

*Note*: Data were assessed for normality by the Kolmogorov–Smirnov test, P–P plot and Q–Q plot and expressed as mean (SD); HC: healthy control; PD: Parkinson disease; PDs: Parkinsonian syndrome; *p*‐values obtained from One‐Way ANOVA adjusted by Bonferroni; T‐αsyn: total α‐syn; P‐αsyn: phosphorylated α‐syn in ser129; Asy‐no: oligomeric α‐syn; PINK1: PTEN‐induced putative kinase 1; P‐TBK1: Phosphorylated TANK‐binding kinase 1; PGAM5: phosphoglycerate mutase 5; BNIP3: BCL2 interacting protein 3.

^a^ Less participants took the examination of three plasma BNIP3 and P‐TBK1 in the modeling cohort (HC = 35, PD = 65, PDs = 54).

^b^ Less participants took the examination of three plasma a‐synuclein levels in the modeling cohort (HC = 49, PD = 90, PDs = 29).

^c^ Participants in validated cohort did not take the examination of plasma T‐αsyn.

### 3.3 Diagnostic accuracy of plasma MAPs biomarkers for PD

Assessing the utility of MAP levels to discriminate between clinically defined idiopathic PD and HC, we found an area under the ROC curve (AUC) of 0.812 (95% CI: 0.759–0.865) for PINK1, 0.786 (95% CI: 0.728–0.834) for Parkin, and 0.827 for PGAM5 (95% CI: 0.771–0.883; Figure 2F). Similarly, ROC tests compared PDs patients against the HC group (Figure 2G), and the AUC for PINK1 was 0.784 (95% CI: 0.718–0.850), which was somewhat lower than for Parkin (AUC 0.810; 95% CI: 0.747–0.873) and higher than PGAM5 (AUC 0.740; 95% CI: 0.668–0.812). Notably, in terms of secondary outcome analysis that compared participants of PD versus PDs (Figure 2H), the AUCs for PINK1 (0.579; 95% CI: 0.504–0.655), Parkin (0.490; 95% CI: 0.414–0.565), and PGAM5 (0.614; 95% CI: 0.537–0.692) were comparable, and all the AUC levels were less than 0.62, indicating the MAPs unable to differentiate the PD from PDs. Regarding the ASPs as a classical diagnosis marker, the AUC values are comparable to the MAPs (Figure S1F–H), indicating MAPs are a good diagnosis marker panel. Next, the incapable discriminative values of plasma p‐TBK1 and BNIP3 in primary and secondary outcomes were displayed in Figure S1I–K, and the corresponding detailed AUC quantitative values in these analyses can be found in Table S1. Then, we want to test if these MAPs can be applied as a compound biomarker to differentiate PD from other neurodegenerative diseases, such as AD. We found an AUC of 0.601 for PINK1, 0.564 for Parkin, and 0.735 for PGAM5 between PD and AD (Figure S1L). The ROC analyses assess the utility of these selected blood biomarker levels to discriminate between AD and PDs (Figure S1N). The AUCs were 0.516 for PINK1, 0.586 for Parkin, and 0.642 for PGAM5.

**FIGURE 3:**
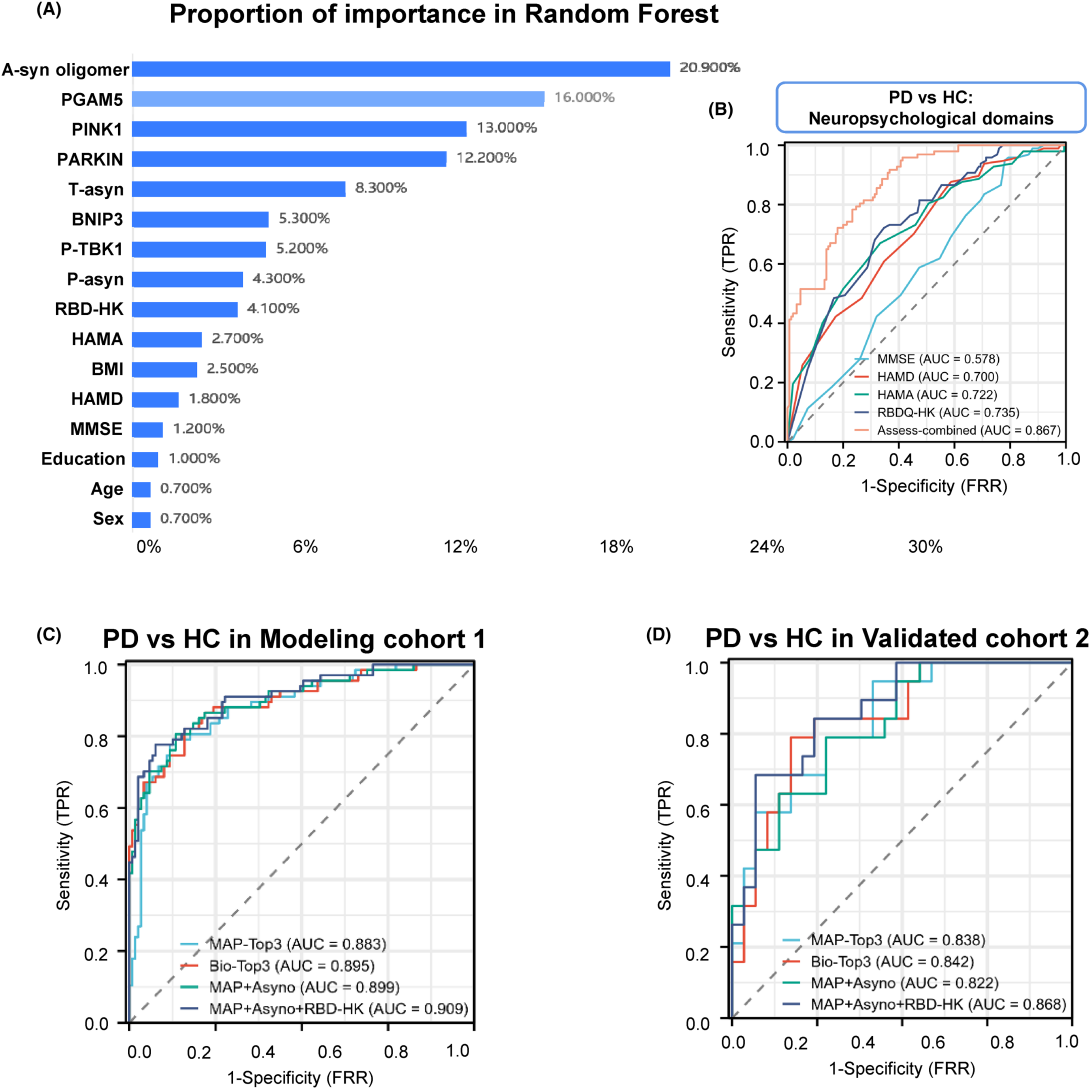
The receiver operating characteristic (ROC) curve analyses the diagnosis accuracy of combined mitophagy‐associated proteins (MAPs) to identify Parkinson disease (PD) in healthy control (HC) participants in the modeling cohort and validated cohort. (A) The random forest can improve the performance of risk predictions based on the decision tree to identify vital features. Rank the important candidates to discriminate PD from HC using the random forest algorithm. (B) Receiver operating characteristic (ROC) curve analyses of neuropsychological domains (Mini‐Mental State Examination, Hamilton Anxiety Scale, Hamilton Depression Scale, and RBD) to differentiate PD from HC. Diagnosis accuracy of combined MAPs to identify PD from HC participants in the modeling cohort (C) and validated cohort (D).

Moreover, machine learning approaches such as random forest classification can improve risk predictions, and using this tool, we found a‐syn oligomer, PGAM5, and PINK1 as the top 3 MAPs discriminating PD from HC (Figure 3A). For RBD, the AUC was 0.735, which was higher than other neuropsychological domains of PD versus HC (e.g., MMSE, HAMD, and HAMA; Figure 3B). Next, we tested whether combining the MAPs separated PD from HC (Figure 3C and Table S2). The AUC was 0.883 (95% CI: 0.831–0.934) when combined with analysis of MAPs (PINK1, PGAM5, and Parkin), which was like the AUCs for Bio‐Top3 (a‐syn oligomer, PINK1 plus Parkin) and MAPs plus a‐syn oligomer of 0.899 (95% CI: 0.85–0.947), indicating the MAPs may be promising as a compound biomarker to differentiate PD from HC. Notably, this observation was corroborated in the validation cohort (Figure 3D).

### 3.4 Association of plasma MAPs biomarkers with motor and nonmotor Features

**FIGURE 4:**
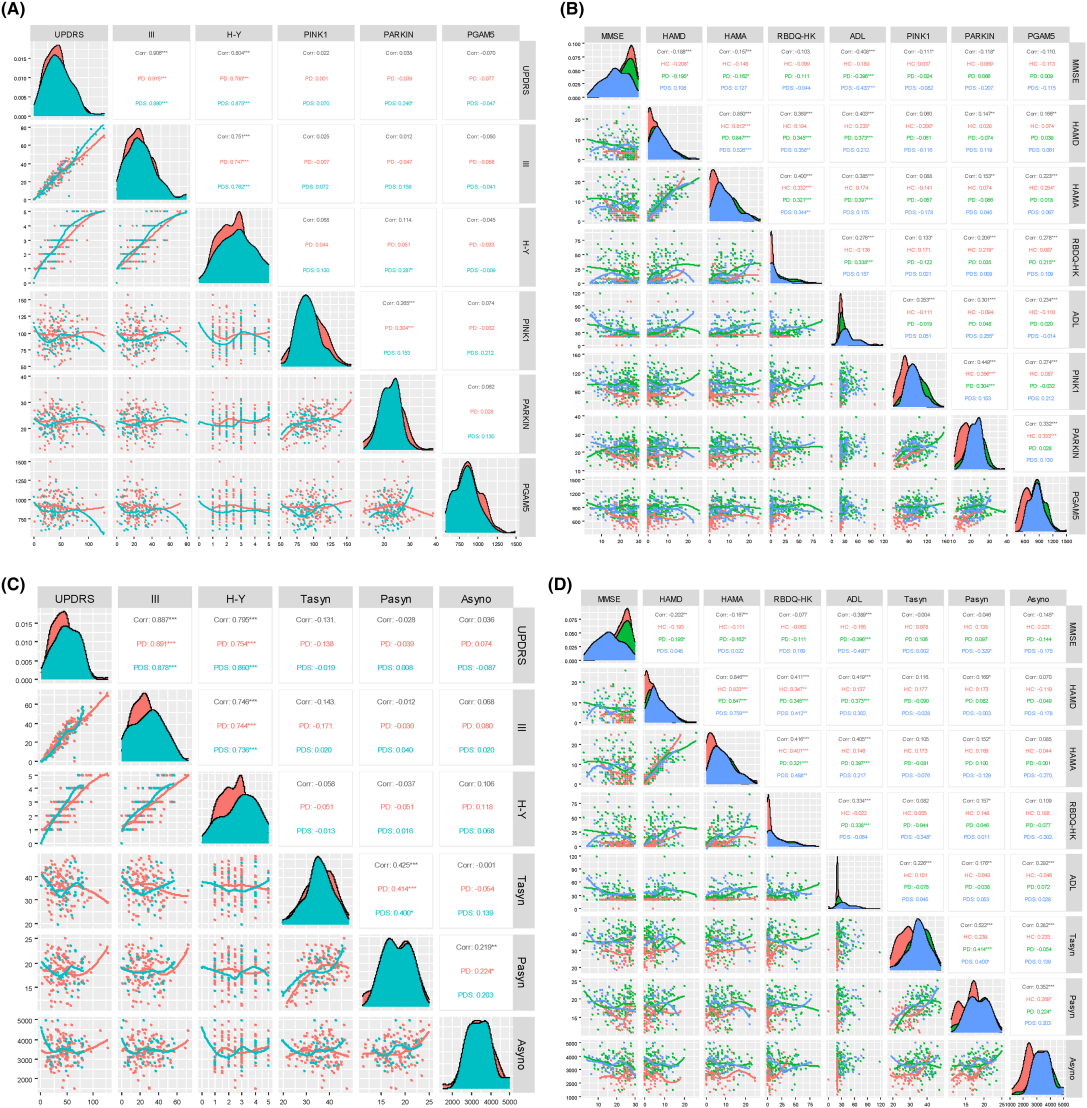
Association of plasma mitophagy‐associated proteins (MAPs) biomarkers with motor and nonmotor features. (A, B) The linear correlation at the cross‐sectional between MAPs (PINK1, Parkin, and PGAM5) and neuropsychological evaluation scales, including total UPDRS, UPDRS Part III, H–Y stage, MMSE, HAMD, HAMA, RBD, and ADL. (C, D) The linear correlation at the cross‐sectional between a‐synuclein‐related proteins and neuropsychological evaluation scales.

We analyzed the correlation between MAP levels (PINK1, Parkin, and PGAM5) and neuropsychological evaluation scales, including total UPDRS, UPDRS Part III, H‐Y stage, MMSE, HAMD, HAMA, RBD, and ADL (Figure 4A,B, and Table S3). Based on the Spearman correlation coefficients (*ρ* values), we found plasma PINK1 positively correlated with RBD (*ρ =* 0.133, *p* = 0.018) and ADL (*ρ =* 0.253, *p* < 0.001) in whole groups and negatively correlated with MMSE (*ρ =* −0.111, *p* = 0.047). Moreover, we then analyzed the correlation between Parkin and motor and nonmotor features in all three groups. Parkin was positively associated with RBD (*ρ =* 0.206, *p* < 0.001) and ADL (*ρ =* 0.301, *p* < 0.001) scores, as well as negatively related to MMSE (*ρ =* −0.118, *p* < 0.035). Additionally, it should be noted that PGAM5 also correlated with the HAMD (*ρ =* 0.166, *p* = 0.003), HAMA (*ρ =* 0.223, *p* < 0.035), RBDQ‐HK (*ρ =* 0.278, *p* < 0.001), and ADL score (*ρ =* 0.234, *p* = 0.001). Next, when examining ASPs as target indexes, the association of plasma ASPs with neuropsychological scales was displayed in Figure 4C,D. In addition, we used non‐linear smoothing spline regressions. RCS models between MAPs as a continuous value and neuropsychological evaluation scales after adjusting for age and sex. Interestingly, we observed no significant positive association between MAPs (PINK1, Parkin, and PGAM5) and UPDRS, MMSE, HAMD, HAMA, and RBD scores in PD subjects (Figure S2 and Table S4). Using MMSE scores as the outcome, RCS analysis revealed an inverse association of Parkin with high MMSE scores and a forward correlation of Parkin with low MMSE scores (Figure S2B). Furthermore, detailed information concern on the association of p‐TBK1, BNIP3, and ASPs with UPDRS, MMSE, HAMD, HAMA, and RBD scores can be found in Figure 3A,B and Figure S4.

### 3.5 Higher plasma MAP levels and prominent diagnostic accuracy in a‐synuclein‐positive subjects

**FIGURE 5:**
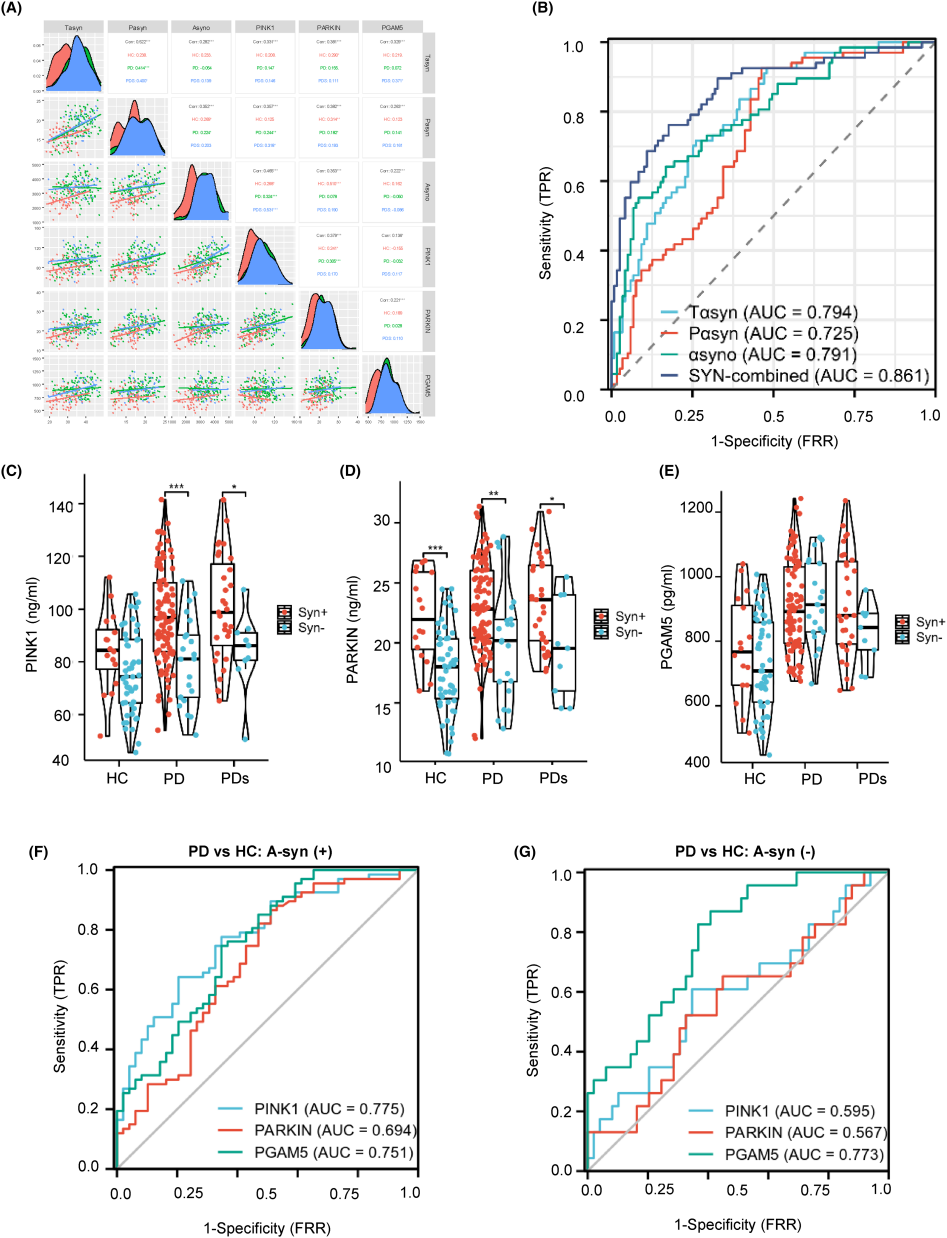
Higher plasma mitophagy‐associated protein (MAP) levels and prominent diagnostic accuracy in a‐synuclein‐positive subjects. (A) The linear correlation at the cross‐sectional between MAPs and a‐synuclein‐related proteins (ASPs). (B) Receiver operating characteristic (ROC) curve analyses of ASPs to get a cutoff value. Levels of PINK1 (C), Parkin (D), and PGAM5 (E) in a‐Syn (+) and a‐Syn (−) subjects. Diagnosis accuracy of plasma PINK1, Parkin, and PGAM5 to differentiate PD from HC in a‐Syn positivity (F) and a‐Syn negativity (G) subjects, respectively, as the reference standard. *p ≤ 0.01, **p ≤ 0.001, ***p ≤ 0.0001.

Being a key hallmark of PD pathogenesis, numerous studies have revealed a‐synuclein as a potential biomarker for PD diagnosis.^23^, ^24^, ^25^ Linear models were applied to evaluate the association between plasma MAPs and ASPs. As shown in Figure 5A, a higher plasma PINK1 level was correlated with higher total a‐syn (*r* = 0.331), phosphorylated a‐syn (*r* = 0.357), and a‐syn oligomer (*r* = 0.465). Similarly, a high level of Parkin was closely related to total a‐syn (*r* = 0.381), phosphorylated a‐syn (*r* = 0.382), and a‐syn oligomer (*r* = 0.353). Plasma PGAM5 was associated with measures of total a‐syn (*r* = 0.329), phosphorylated a‐syn (*r* = 0.263), and a‐syn oligomer (*r* = 0.222). Hence, to test the MAP levels and diagnostic accuracy in the a‐syn‐positive subjects. A‐syn distinguishes a‐Syn (+) from a‐Syn (−) by the cutoff value obtained from PD and HC groups by a logistic regression model using total a‐syn, phosphorylated a‐syn, and a‐syn oligomer (Figure 5B). We found abnormally higher PINK1 (HC: 84.2 ± 15.0 vs. 76.0 ± 15.1 ng/mL; PD: 97.6 ± 17.8 vs. 80.6 ± 16.7 ng/mL, respectively) and Parkin (HC: 22.2 ± 3.68 vs. 17.7 ± 3.53 ng/mL; PD: 23.0 ± 3.72 vs. 20.1 ± 4.72 ng/mL, respectively) levels in the a‐Syn (+) subjects compared to a‐Syn (−) populations (Figure 5C–E and Table S5). Of interest, the levels of p‐TBK1 were also higher in the a‐Syn (+) than a‐Syn (−) subjects, but not BNIP3 (Figure S3C,D). Moreover, comparing PD versus CN, plasma PINK1 level discriminated abnormal versus normal a‐synuclein status with an AUC of 0.775 in a‐syn (+) versus 0.595 in a‐syn (−) subjects (Figure 5F,G), which was mild higher than the AUCs for plasma levels of Parkin (0.694 vs. 0.567) and PGAM5 (0.751 vs. 0.773), indicating PINK1 showed higher diagnostic accuracy than established plasma Parkin and PGAM5 biomarkers in A‐syn (+) subjects. See Figure S3E, f for the AUCs of p‐TBK1 and BNIP3 in the A‐syn (+) or (−) subjects.

### 3.6 Correlation and concordance among plasma MAP levels

To investigate the relationship and concordance between plasma PINK1, Parkin, and PGAM5. A linear correlation analysis model displayed that plasma PINK1 was sensitive to the Parkin level (*r* = 0.409, *p* < 0.001, Figure S5A). Notably, the relationship between plasma PINK1 and PGAM5 was mild and low *r* value (*r* = 0.127, *p* = 0.073, see Figure S5B). Of interest, for the biomarkers Parkin and PGAM5, we found that plasma Parkin was concordant with the PGAM5 distribution (*r* = 0.158, *p* = 0.025, see Figure S5C). These results indicate that plasma PINK1, Parkin, and PGAM5 have similar and related expression trajectories.

## 4 DISCUSSION

To the best of our knowledge, this is a full‐scale study of such a topic in the PD biomarker fields, and we detail baseline plasma MAP (e.g., PINK1, Parkin, and PGAM5) levels of PD and PDs compared with healthy controls. We found that (1) Subjects with PD or PDs exhibit higher MAP levels compared to healthy controls. (2) The AUCs of PINK1, PGAM5, and Parkin were ranked as the top three MAPs differentiating PD from HC. (3) The MAPs could not differentiate PD from PDs. (4) Higher plasma PINK1‐Parkin levels and prominent diagnostic accuracy in A‐syn (+) subjects than in A‐syn (−) subjects. Thus, we pinpointed plasma PINK1, PGAM5, and Parkin as promising candidate MAP biomarkers of the target mitophagy process in PD.

Although numerous studies have described the potential role of mitophagy in PD pathogenesis according to basic research,^26^, ^27^ few reports have assessed MAP levels in biofluids from PD cases. Our data are in line with the theory that patients with PD exhibit higher MAPs due to impaired mitochondrial function, which induces mitophagy. However, here we found that at the disease stage with cognitive impairment (such as MMSE scores <21), the Parkin levels lean to decline with the deterioration of cognition, revealing an inverted U‐shaped curve between Parkin and PD cognitive symptom severity. Furthermore, the potential of PINK1, PGAM5, and Parkin as biomarkers were strengthened by our finding that these markers discriminated PD from control in two independent cohorts, even though the sample size was relatively small in the second cohort. Notably, although the p‐TBK1 and BNIP3 are involved in the mitophagy process,^28^, ^29^ we found that the levels of these two markers were not different among these three groups, indicating the complexity of the mitophagy pathway.

Previous works identified mitochondrial dysfunction and impaired mitophagy as associated with PD pathogenesis.^9^ This theory is supported by several animal experiments, where similar Parkinsonian features were detected in MAPs knockout animals, including *PINK1* ^−/−^, *Parkin* ^−/−^, and *PGAM5* ^−/−^.^12^, ^30^, ^31^ Recently, emerging evidence has focused on the relationship between mitophagy and PD genes, such as the LRRK2 mutations^32^ and observations that α‐synuclein interacts with outer mitochondrial membrane substrates, disrupting the mitophagy pathway.^33^ Biochemical studies in transfected cells and transgenic mice suggest that PINK1 and Parkin act in concert with a mitochondrial quality control system in neuroprotection.^10^, ^34^ Correspondingly, Mendelian genetics attributes loss‐of‐function mutations in PINK1 and Parkin, two key mitophagy regulators, to early‐onset PD.^12^ Based on such critical findings, we want to test the role of MAPs in the PD diagnosis domain. As mentioned earlier, we have previously speculated that MAPs could be increased in the PD background due to the impaired mitochondria leading to inverse feedback. More than that, the link between impaired mitophagy and PD has opened a window of opportunity for multiple therapeutics targeting mitochondrial quality control. The regimen of interventions ranges from boosting mitophagy activity via specific activators to mitophagy pathway‐related gene therapy.

The observed close association of the PGAM5 in this cohort with clinical scales commonly utilized to assess the severity of nonmotor symptoms may be explained for several potential reasons. It is possible that the PGAM5 levels are probably reactive or coincidental in more severely progressing individuals. Alternatively, the PGAM5 values measured in plasma may reflect part of the PD process driving more severe disease in some individuals. Conversely, there was no association between the PINK1/PGAM5 and the MMSE score, indicating that cognitive dysfunction in PD patients is not related to the reduction of the PINK1 and PGAM5 levels. However, p‐TBK1 and BNIP3 did not show a tight relationship with the clinical parameters. This might reflect functional heterogeneity between different MAPs in the mitophagy process that is not well understood. Because Parkin levels were higher in PD or PDs than in controls, we posit that Parkin may play a disparate role in the mitophagy pathway compared to PINK1 and disease feature progression.^35^ Of note, the clinical parameters in this study also have some limitations, including sensitivity and assessment accuracy, etc. Moreover, further investigations in larger cohorts with biochemical and advanced measurement tools may be revealing in this regard. More importantly, studies examining MAP levels in the CSF in addition to plasma may firmly test its relevance as a disease biomarker for PD.

Moreover, as mentioned, PD is closely related to a‐synuclein mutations, which are located in mitochondria and lead to mitochondrial dysfunction, block normal mitochondrial clearance, and lead to the accumulation of damaged mitochondria in the body.^36^ To maintain homeostasis, the body activates the PINK1/Parkin mitophagy pathway, which leads to an increase in plasma mitochondrial protein levels. In addition, we do not know yet whether this pattern exists in CSF or other body fluids, and further research is needed to explore this issue. In terms of other typical neurodegenerative diseases, mitophagy is also impaired in AD, amyotrophic lateral sclerosis (ALS), Huntington's disease (HD), and frontotemporal dementia (FTD). Disorganized mitophagy in postmortem brain samples from AD subjects and in AD models is caused by several mechanisms, including by the inhibition of the ULK1/TBK1‐dependent initiation of the mitophagic machinery via tau/Aβ proteinopathies.^37^ A study carrying HeLa cells with protein overexpression indicated that insufficient turnover and aggregates of injured mitochondria may lead to disease progression in ALS.^38^ Several genes linked to ALS and FTD encode proteins involved in mitophagy, including optineurin (OPTN), TANK‐binding kinase 1 (TBK1), p62, and receptor‐interacting protein kinase 1 (PIPK1) et al,^10^ but how mutation of these genes contributes to the pathology is not fully understood. For HD issues, damaged mitochondrial and proteostasis alterations, especially in the autophagic/endosomal system, including the mutation of Autophagy Related Protein 7 (ATG7) and impairment of GAPDH‐mediated mitophagy by mutant Htt, are related to the pathogenesis of HD.^39^ In summary, it is likely that impaired mitophagy is a common condition in many neurodegenerative pathologies.

Interestingly, we also found increased MAP levels in A‐syn (+) subjects compared to A‐syn (−) subjects, and we provide here the first evidence for a significant augment of diagnosis capacity in A‐syn‐positive patients, supporting the hypothesis that MAP levels or the mitophagy process closely interact with a‐synuclein aggregation in this disease. The increase in plasma MAP levels detected in the A‐syn (+) background may be related to more serious mitochondrial dysfunction. The loss of mitochondrial dysfunction and the appearance of Lewy bodies (A‐syn aggregation) are two pathological features of PD. Under mild to moderate mitochondrial stress conditions, PINK1, Parkin, and a‐synuclein form a regulatory circuit to manage the mitochondrial stress response.^40^ Moreover, Xiaoxi et al reported that human a‐syn A53T overexpression in transgenic mice induces pervasive mitophagy impairment preceding dopaminergic neuron degeneration. They found that mitochondria hubs are the main targets of a‐syn and defective mitophagy plays a vital role during disease pathogenesis,^41^ indicating the MAPs interaction with a‐syn tightly and offering the reason behind the higher MAP levels in A‐syn (+) subjects.

Despite some interesting observations on differences in MAP signatures in PD and PDs, our project still has some limitations. This study employed a relatively low‐throughout way of centrifugation of blood biospecimens and a laborious enzyme‐linked immunosorbent assay (ELISA) to analyze the target proteins. This assay is often performed manually and is therefore difficult to standardize. Moreover, the measurement of plasma biomarkers was done with ELISA assays not known in the field, alluding to the precision issue. Higher‐throughput assays with increased sensitivity may provide additional insights. For instance, single‐molecule‐based assays (Simoa) or real‐time quaking‐induced conversion (RT‐QuIC) have been successfully developed for ultralow concentrations of proteins in biofluids,^42^, ^43^ they have been shown to be more sensitive than traditional immunoassays. Furthermore, given the effect of storage time on the MAPs expression in our serum samples, it needs attention that future studies should carefully control for this variation and take it into consideration when analyzing the data.

## 5 CONCLUSIONS

In summary, we show that plasma MAPs (PINK1, PGAM5, and Parkin) may be potentially useful biomarkers for PD diagnosis, and PINK1 reflects disease symptom progression closely. Studies on larger cohorts would be required to test whether elevated plasma MAP levels are related to PD risk or prediction.

## AUTHOR CONTRIBUTIONS

Haijun He, Wenwen Wang, Huimin Zhu, and Shuangjie Qian prepared the samples and analyzed the data. Xi Xiong and Qianqian Ye recruited the participants, and Shuoting Zhou made substantial contributions to conception and replenished the required data. Hilde Nilsen and Chenglong Xie were involved in drafting the manuscript.

## FUNDING INFORMATION

Supported by the Projects of the National Science Foundation of China (No. 81600977 and 82271469) and the Projects of the Natural Science Foundation of Zhejiang Province (LQ23H090007, Y19H090059, and LZ23H090001).

## CONFLICT OF INTEREST STATEMENT

No commercial or financial relationship could be construed as a potential conflict of interest.

## DISCLOSURES

The authors report no disclosures relevant to the manuscript.

## Supporting information


Figure S1.
Figure S2.
Figure S3.
Figure S4.
Figure S5.
Table S1.
Table S2.
Table S3.
Table S4.
Table S5.

## Data Availability

The data that support the findings of this study are available on request from the corresponding author. The data are not publicly available due to privacy or ethical restrictions.
