# Supplementary material for: Identification of mitophagy‐associated proteins profile as potential plasma biomarkers of idiopathic Parkinson's disease
Source: CNS Neurosci Ther. 2023 Nov 21;30(4):e14532. doi: 10.1111/cns.14532 (PMC11056850; doi:10.1111/cns.14532)
Supplement: Supplementary file 1 — Figure S1. Figure S2. Figure S3. Figure S4. Figure S5. Table S1. Table S2. Table S3. Table S4. Table S5. [file CNS-30-e14532-s001.pdf]

Supplementary documents

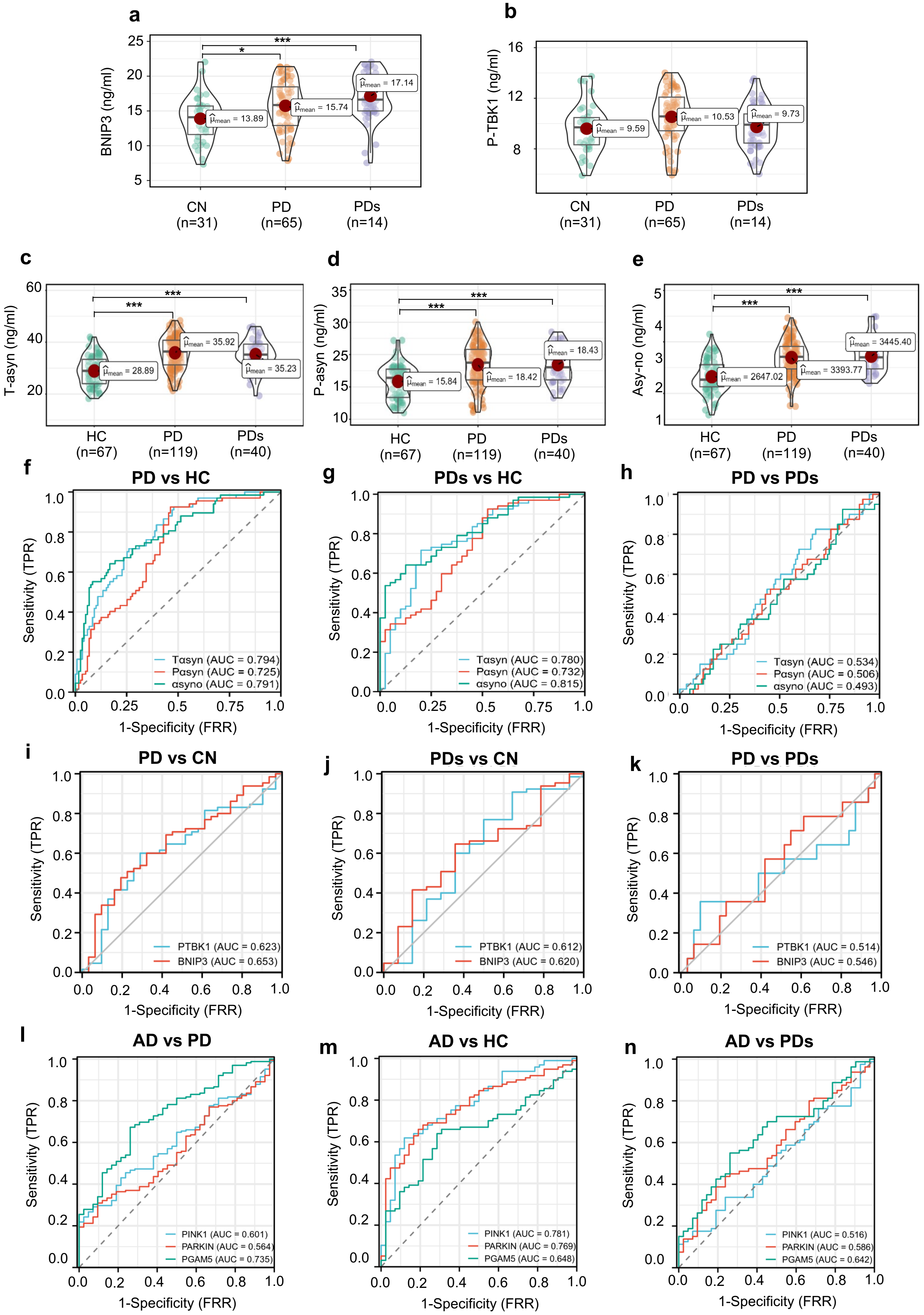

Supple Figure 1

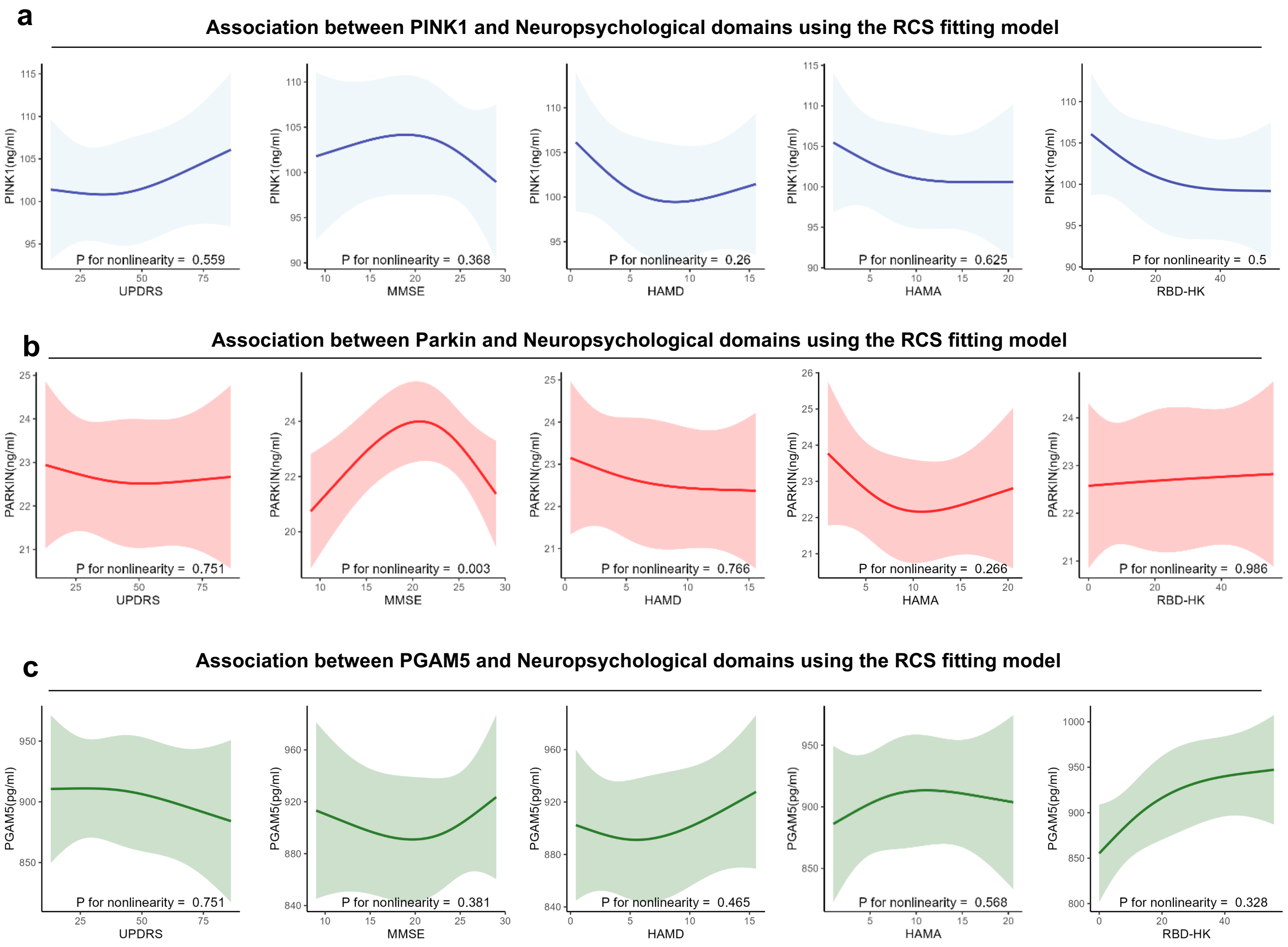

Supple Figure 2

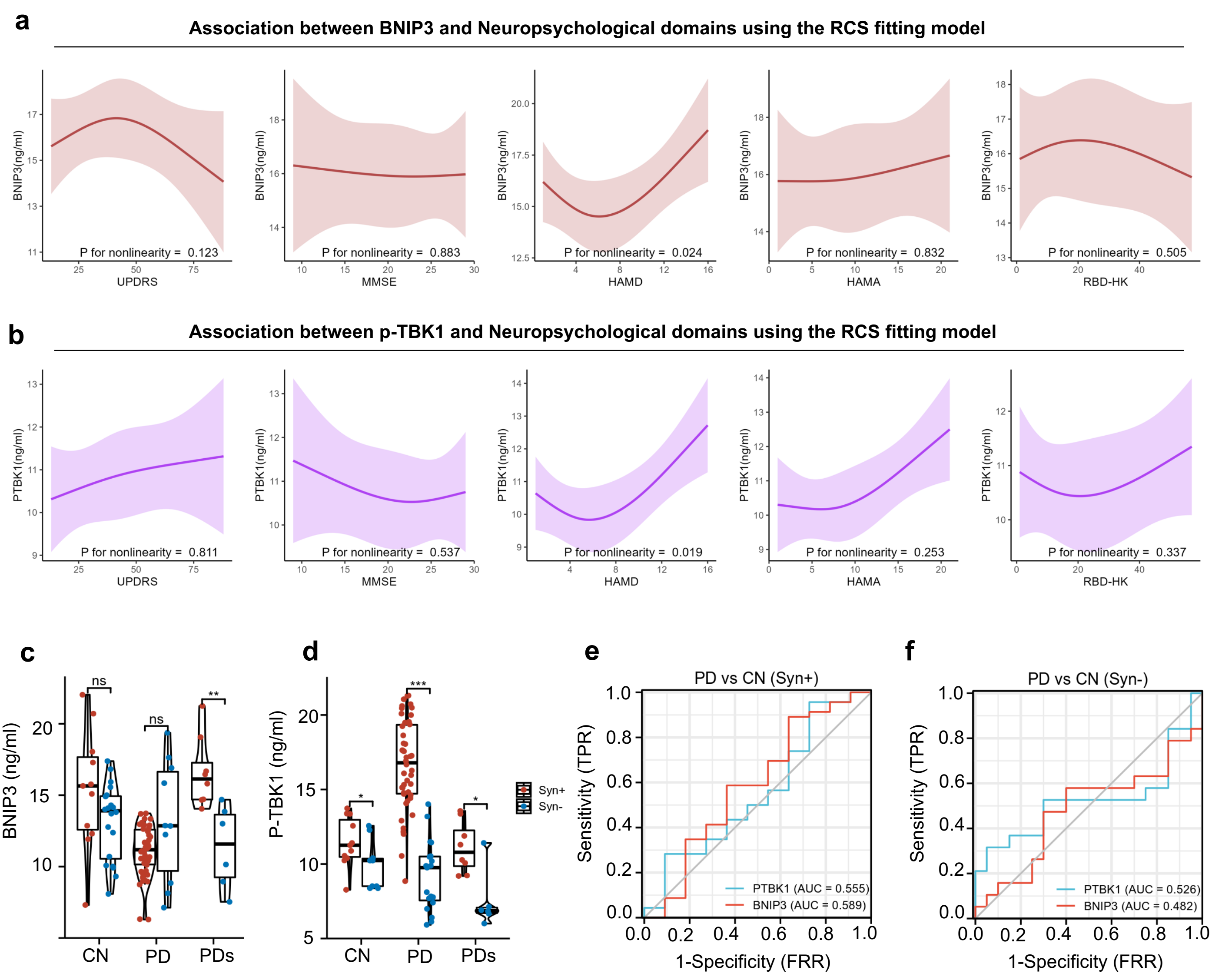

Supple Figure 3

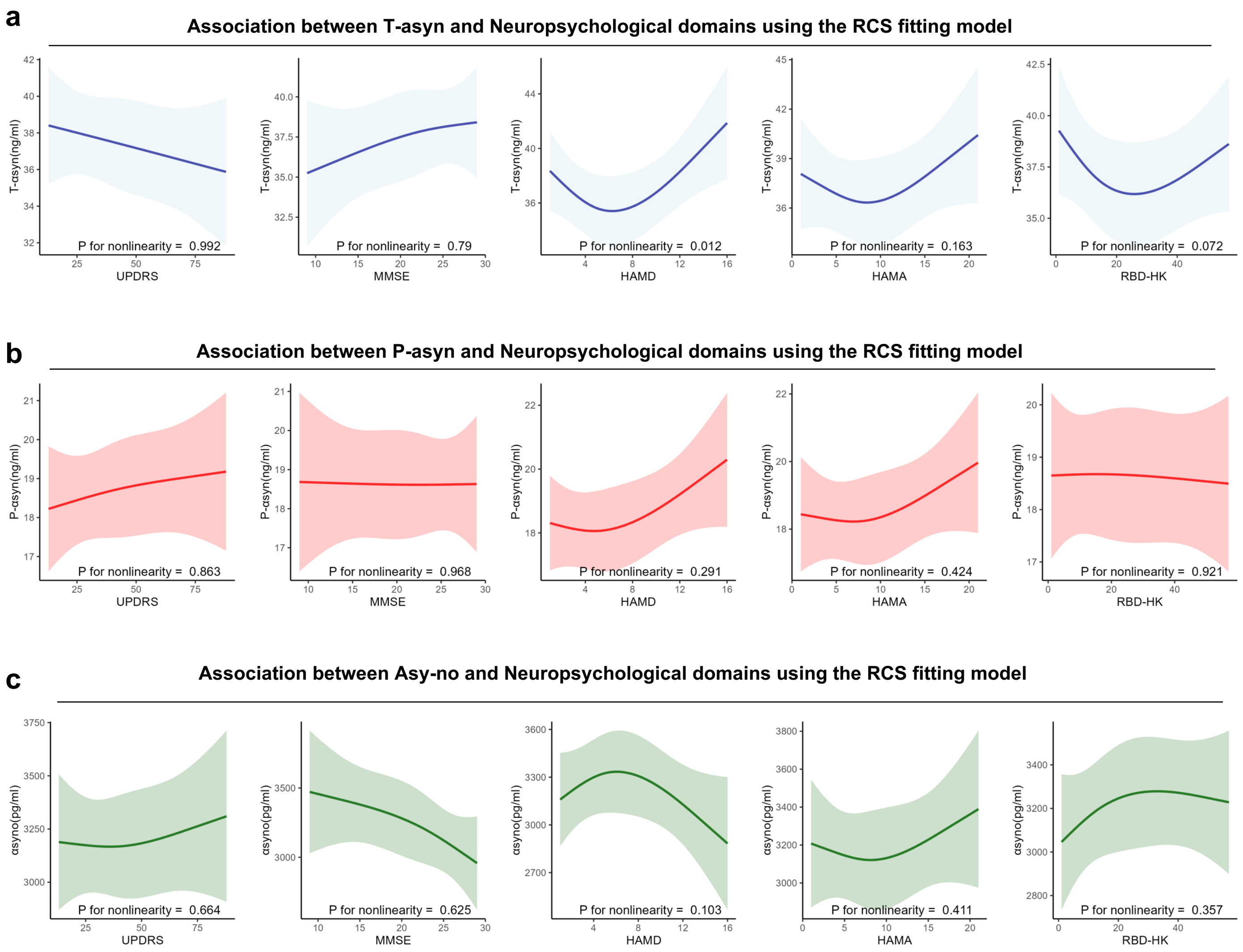

Supple Figure *4*

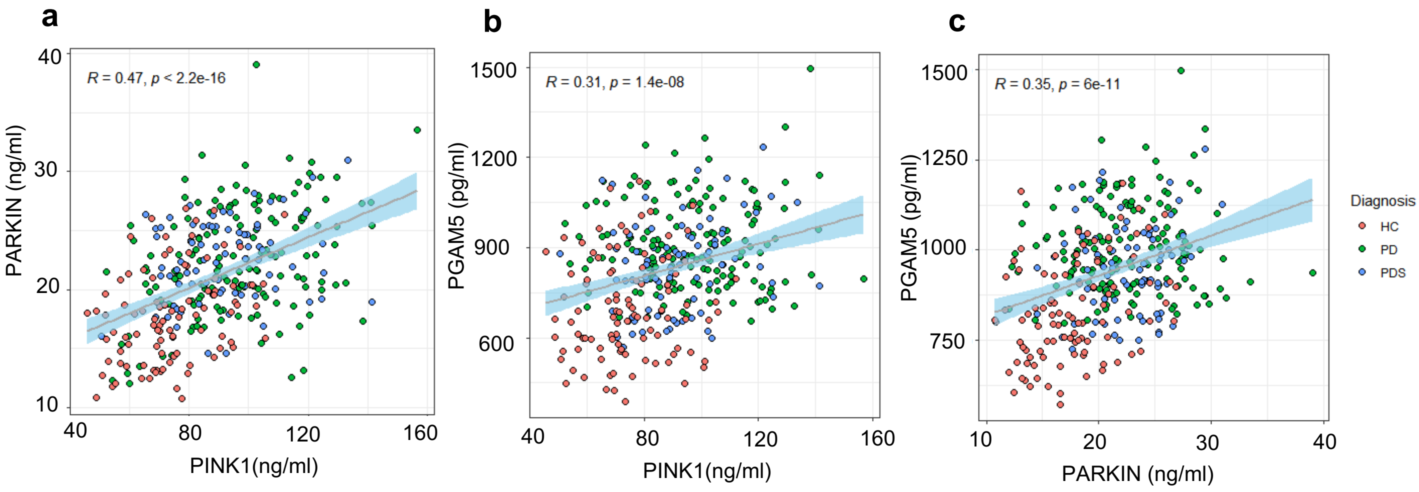

Supple Figure 5

Supplementary Table 1: Diagnostic performance of different plasma-derived multiple biomarker levels in differentiating PD, PDs, and HC

Features	AUC [95% CI]		
	PD vs HC	PDs vs HC	PD vs PDs
PINK1	0.812 [0.759, 0.865]	0.784 [0.718, 0.850]	0.579 [0.504, 0.655]
PARKIN	0.786 [0.728, 0.843]	0.81 [0.747, 0.873]	0.49 [0.414, 0.565]
PGAM5	0.827 [0.771, 0.883]	0.74 [0.668, 0.812]	0.614 [0.537, 0.692]
BNIP3	0.649 [0.535, 0.762]	0.533 [0.408, 0.658]	0.629 [0.528, 0.730]
P-TBK1	0.657 [0.546, 0.767]	0.767 [0.664, 0.870]	0.613 [0.511, 0.714]
T-αsyn	0.794 [0.730, 0.858]	0.78 [0.688, 0.873]	0.534 [0.434, 0.635]
P-αsyn	0.725 [0.652, 0.798]	0.732 [0.634, 0.831]	0.506 [0.404, 0.608]
Asy-no	0.791 [0.723, 0.859]	0.815 [0.737, 0.893]	0.493 [0.390, 0.597]
MMSE	0.578 [0.507, 0.649]	0.767 [0.694, 0.840]	0.673 [0.599, 0.747]
HAMD	0.700 [0.635, 0.766]	0.704 [0.624, 0.784]	0.504 [0.424, 0.585]
HAMA	0.722 [0.657, 0.787]	0.7 [0.621, 0.779]	0.463 [0.383, 0.543]
RBDQ-HK	0.735 [0.673, 0.797]	0.59 [0.502, 0.677]	0.637 [0.558, 0.715]

Data are presented as AUC [95% CI] (DeLong statistics) in differentiating PD vs HC, PD vs PDs and PDs vs HC; AUC, area under the receiver operating characteristic curve; CI, confidence interval; OR, odds ratio; Other abbreviations as in table 1 and table 2.

Supplementary Table 2. Comparisons of the accuracy for combined models in distinguishing PD and HC

Model type	Variables in model	OR [95% CI]	AUC [95% CI]	P-value	AIC
**1. MAP-Top3**	PINK1	1.056 [1.033, 1.082]	0.883 [0.831, 0.934]	0.318 vs 2	185.37
	PARKIN	1.107 [1.014, 1.214]		0.156 vs 3	
	PGAM5	1.008 [1.005, 1.011]		0.056 vs 4	
**2. Bio-Top3**	PINK1	1.054 [1.027, 1.085]	0.895 [0.846, 0.945]	0.318 vs 1	147.74
	PGAM5	1.009 [1.006, 1.013]		0.303 vs 3	
	αsyno	1.001 [1.001, 1.002]		0.110 vs 4	
**3. MAP + Asy-no**	PINK1	1.048 [1.019, 1.081]	0.899 [0.85, 0.947]	0.156 vs 1	148.79
	PARKIN	1.056 [0.947, 1.18]		0.303 vs 2	
	PGAM5	1.009 [1.006, 1.013]		0.177 vs 4	
	αsyno	1.001 [1.001, 1.002]			
**4. MAP + Asy-no + RBDQ-HK**	PINK1	1.051 [1.022, 1.086]	0.909 [0.864, 0.954]	0.056 vs 1	142.54
	PARKIN	1.032 [0.924, 1.156]		0.110 vs 2	
	PGAM5	1.009 [1.005, 1.013]		0.177 vs 3	
	αsyno	1.001 [1.000, 1.002]			
	RBDQ-HK	1.044 [1.012, 1.084]			

Results are from logistic regression models using PD vs HC as the outcome. *P*-values are from the comparison of AUCs (DeLong statistics). Abbreviations: MAP: Mitophagy-associated protein; AIC, Akaike information criterion (lower equals a better model fit); AUC, area under the receiver operating characteristic curve; CI, confidence interval; OR, odds ratio. Other abbreviations as in Table 1 and Table 2.

Supplementary Table 3: Correlation of different plasma-derived biomarker levels with motor and nonmotor features

ρ (*p*)	UPDRS	III	H-Y stage	MMSE	HAMD	HAMA	RBDQ-HK	ADL
**ALL**								
PINK1	0.022 (0.742)	0.025 (0.707)	0.068 (0.312)	-0.111 (0.047)	0.06 (0.285)	0.088 (0.115)	0.133 (0.018)	0.253 (<0.001)
PARKIN	0.038 (0.576)	0.012 (0.864)	0.114 (0.089)	-0.118 (0.035)	0.147 (0.008)	0.153 (0.006)	0.206 (<0.001)	0.301 (<0.001)
PGAM5	-0.07 (0.297)	-0.05 (0.459)	-0.045 (0.508)	-0.11 (0.05)	0.166 (0.003)	0.223 (<0.001)	0.278 (<0.001)	0.234 (<0.001)
PTBK1	0.055 (0.567)	0.008 (0.93)	0.043 (0.652)	-0.005 (0.957)	0.152 (0.067)	0.19 (0.022)	0.141 (0.089)	0.139 (0.095)
BNIP3	-0.052 (0.583)	-0.078 (0.416)	-0.009 (0.922)	-0.071 (0.397)	0.148 (0.074)	0.055 (0.512)	-0.015 (0.852)	0.245 (0.003)
T-αsyn	-0.131 (0.099)	-0.143 (0.073)	-0.058 (0.465)	-0.004 (0.951)	0.116 (0.083)	0.105 (0.117)	0.082 (0.219)	0.226 (0.001)
P-αsyn	-0.028 (0.724)	-0.012 (0.881)	-0.037 (0.642)	-0.046 (0.489)	0.169 (0.011)	0.152 (0.022)	0.157 (0.018)	0.176 (0.008)
αsyno	0.036 (0.657)	0.068 (0.395)	0.106 (0.185)	-0.145 (0.03)	0.07 (0.293)	0.085 (0.201)	0.109 (0.103)	0.292 (<0.001)
HC								
PINK1	-	-	-	0.037 (0.718)	-0.2 (0.05)	-0.141 (0.167)	0.171 (0.094)	-0.111 (0.279)
PARKIN	-	-	-	-0.069 (0.504)	0.028 (0.788)	0.074 (0.471)	0.219 (0.032)	-0.094 (0.362)
PGAM5	-	-	-	-0.113 (0.27)	0.074 (0.473)	0.254 (0.012)	0.087 (0.395)	-0.11 (0.285)
PTBK1	-	-	-	-0.141 (0.42)	0.036 (0.838)	0.013 (0.94)	0.169 (0.333)	0.088 (0.617)
BNIP3	-	-	-	0.06 (0.73)	0.132 (0.448)	0.011 (0.951)	0.028 (0.872)	0.117 (0.503)
T-αsyn	-	-	-	0.078 (0.529)	0.177 (0.153)	0.173 (0.161)	0.055 (0.658)	0.101 (0.417)
P-αsyn	-	-	-	0.135 (0.275)	0.173 (0.163)	0.169 (0.171)	0.148 (0.232)	-0.043 (0.728)
αsyno	-	-	-	0.221 (0.072)	-0.119 (0.337)	-0.044 (0.722)	0.188 (0.128)	-0.046 (0.713)
PD								
PINK1	0.001 (0.987)	-0.007 (0.932)	0.044 (0.592)	-0.024 (0.773)	-0.061 (0.456)	-0.057 (0.489)	-0.122 (0.136)	-0.019 (0.82)
PARKIN	-0.039 (0.64)	-0.047 (0.567)	0.051 (0.535)	0.066 (0.419)	-0.074 (0.365)	-0.086 (0.297)	0.035 (0.668)	0.048 (0.558)
PGAM5	-0.077 (0.35)	-0.058 (0.479)	-0.033 (0.692)	0.009 (0.917)	0.039 (0.637)	0.018 (0.827)	0.215 (0.008)	0.02 (0.808)
PTBK1	0.092 (0.464)	0.067 (0.594)	0.04 (0.749)	0.031 (0.809)	0.21 (0.094)	0.315 (0.011)	0.034 (0.788)	0.13 (0.303)
BNIP3	0.002 (0.987)	-0.044 (0.727)	-0.071 (0.576)	-0.082 (0.518)	0.157 (0.211)	0.102 (0.421)	-0.035 (0.781)	-0.003 (0.979)
T-αsyn	-0.138 (0.133)	-0.171 (0.062)	-0.051 (0.582)	0.106 (0.253)	-0.09 (0.332)	-0.081 (0.38)	-0.044 (0.634)	-0.078 (0.397)
P-αsyn	-0.039 (0.674)	-0.03 (0.742)	-0.051 (0.581)	0.097 (0.295)	0.082 (0.373)	0.1 (0.28)	0.046 (0.622)	-0.038 (0.682)
αsyno	0.074 (0.426)	0.08 (0.388)	0.118 (0.203)	-0.144 (0.117)	-0.049 (0.6)	-0.001 (0.995)	-0.077 (0.408)	0.072 (0.438)
PDs								
PINK1	0.07 (0.555)	0.072 (0.547)	0.13 (0.274)	-0.082 (0.496)	-0.116 (0.333)	-0.178 (0.135)	0.021 (0.862)	0.051 (0.67)
PARKIN	0.24 (0.041)	0.159 (0.18)	0.287 (0.014)	-0.207 (0.081)	0.119 (0.32)	0.045 (0.707)	0.009 (0.942)	0.255 (0.031)
PGAM5	-0.047 (0.694)	-0.041 (0.731)	-0.009 (0.938)	-0.115 (0.337)	0.061 (0.613)	0.067 (0.573)	0.109 (0.358)	-0.014 (0.906)
PTBK1	0.037 (0.804)	-0.011 (0.942)	0.151 (0.311)	-0.05 (0.74)	-0.021 (0.89)	-0.036 (0.814)	-0.022 (0.881)	0.164 (0.277)

BNIP3	-0.145 (0.332)	-0.149 (0.319)	0.02 (0.894)	0.072 (0.637)	-0.075 (0.622)	-0.275 (0.065)	-0.068 (0.651)	0.26 (0.081)
T-αsyn	-0.019 (0.906)	0.02 (0.902)	-0.013 (0.939)	0.002 (0.992)	-0.028 (0.863)	-0.076 (0.641)	-0.348 (0.028)	0.045 (0.783)
P-αsyn	0.008 (0.963)	0.04 (0.808)	0.016 (0.923)	-0.329 (0.038)	-0.003 (0.987)	-0.129 (0.426)	0.011 (0.946)	0.053 (0.743)
αsyno	-0.087 (0.593)	0.02 (0.903)	0.068 (0.676)	-0.175 (0.281)	-0.178 (0.271)	-0.27 (0.092)	-0.302 (0.058)	0.028 (0.865)

Data are expressed as *ρ* (*p* value) achieved by spearman correlation. Abbreviations as in table 1 and table 2.

Supplementary Table 4: *P* value for nonlinearity using RCS models

***p* for nonlinearity**	**PINK1**	**PARKIN**	**PGAM5**	**BNIP3**	**P-TBK1**	**T-αsyn**	**P-αsyn**	**Asy-no**
UPDRS	0.559	0.751	0.751	0.124	0.811	0.500	0.730	0.829
MMSE	0.368	0.003	0.381	0.881	0.535	0.673	0.383	0.541
HAMD	0.260	0.766	0.465	0.024	0.019	0.017	0.102	0.823
HAMA	0.625	0.266	0.568	0.821	0.246	0.053	0.263	0.807
RBDQ-HK	0.500	0.986	0.328	0.503	0.335	0.144	0.523	0.724
ADL	0.644	0.524	0.127	0.226	0.493	0.459	0.339	0.687

Correlations between biomarkers and neuropsychological domains in PD patients using the Restricted cubic spline curves (RCS) fitting model. P for nonlinearity was obtained in each RCS model.

Supplementary Table 5: Demographic characteristics and biomarker levels of different α-syn status in Modeling cohort

**A-syn status**	**A-syn (+)**			**A-syn (-)**		
**Characteristics**	**HC (N = 16)**	**PD (N = 98)**	**PDs (N = 31)**	**HC (N = 51)**	**PD (N = 21)**	**PDs (N = 9)**
Age (years)	67.5 [64.8;72.5]	66.0 [60.0;71.0]	70.0 [64.0;75.0]	64.0 [57.5;69.0]	66.0 [62.0;70.0]	60.0 [57.0;66.0]
Female (%)	9 (56.2%)	47 (48.0%)	13 (41.9%)	32 (62.7%)	6 (28.6%)	4 (44.4%)
Height (cm)	159 (5.37)	160 (7.59)	162 (8.32)	162 (6.69)	163 (8.68)	158 (10.6)
Weight (kg)	61.5 (6.60)	60.6 (9.84)	60.9 (9.83)	62.4 (9.60)	65.8 (12.3)	65.0 (10.3)
BMI	24.2 (2.76)	23.6 (3.05)	23.3 (3.36)	23.8 (2.91)	24.7 (3.73)	26.6 (6.55)
Education (years)	5.00 [2.25;6.00]	4.00 [0.00;6.00]	5.00 [0.50;6.50]	4.00 [0.00;6.00]	5.00 [0.00;8.00]	2.50 [0.00;6.00]
Disease History (years)	-	4.00 [1.00;7.00]	2.00 [1.00;4.50]	-	3.00 [2.00;4.00]	1.50 [1.00;3.00]
UPDRS	-	40.0 [26.5;53.0]	49.0 [34.0;69.0]	-	42.0 [24.0;52.0]	49.0 [38.0;65.0]
I	-	2.00 [1.00;4.00]	3.00 [2.00;5.00]	-	1.00 [0.00;3.00]	4.00 [2.00;4.00]
II	-	10.0 [6.00;16.0]	13.0 [10.0;18.5]	-	10.0 [8.00;15.0]	13.0 [11.0;17.0]
III	-	25.0 [16.0;33.8]	32.0 [16.0;41.5]	-	23.0 [16.0;40.0]	29.0 [19.0;36.0]
IV	-	1.00 [0.00;4.00]	1.00 [0.00;2.00]	-	2.00 [1.00;3.00]	0.00 [0.00;3.00]
H-Y stage	-	2.50 [1.50;3.00]	3.00 [2.00;4.00]	-	2.50 [1.50;3.00]	3.00 [1.50;4.00]

MMSE	26.0 [23.8;27.0]	23.0 [18.0;26.0]	15.0 [9.50;18.5]	23.0 [20.0;26.0]	25.0 [18.0;27.0]	19.0 [16.0;24.0]
HAMD	3.50 [0.75;5.00]	4.00 [2.00;9.00]	5.00 [3.00;9.00]	3.00 [0.50;5.00]	5.00 [2.00;7.00]	5.00 [5.00;14.0]
HAMA	5.00 [2.75;9.25]	7.50 [4.00;12.8]	6.00 [4.00;11.0]	4.00 [1.00;7.00]	6.00 [4.00;11.0]	9.00 [8.00;14.0]
RBDQ-HK	3.50 [1.75;16.2]	13.0 [3.00;30.0]	4.00 [1.50;22.0]	3.00 [1.00;8.00]	13.0 [4.00;41.0]	27.0 [15.0;31.0]
ADL	20.0 [20.0;20.0]	25.5 [21.0;34.8]	37.0 [25.5;56.0]	20.0 [20.0;20.0]	27.0 [20.0;34.0]	34.0 [31.0;36.0]
PINK1 (ng/mL)	84.2 (15.0)	97.6 (17.8)***	100 (20.3) *	76.0 (15.1)	80.6 (16.7)	83.9 (15.3)
Parkin (ng/mL)	22.2 (3.68)***	23.0 (3.72)**	23.4 (3.70)*	17.7 (3.53)	20.1 (4.72)	19.6 (4.19)
PGAM5 (pg/mL)	778 (159)	909 (138)	916 (160)	730 (145)	919 (126)	823 (92.6)
BNIP3 (ng/mL)	11.5 (1.67)	11.1 (1.76)***	11.1 (1.61)**	8.87 (1.36)	8.85 (2.11)	6.71 (0.43)
P-TBK1 (ng/mL)	15.4 (4.20)***	16.6 (2.98)***	16.4 (2.38)***	13.1 (2.65)	13.3 (4.02)	10.7 (2.68)

Data are presented as mean (SD) or number (percentage) as appropriate. *p*-values obtained from Mann-Whitney U test for continuous variables or chi-squared test for categorical variables. HC: Healthy Control; PD: Parkinson disease; PDs: Parkinsonian syndrome; UPDRS: unified Parkinson's disease rating scale; MMSE: Mini-Mental State Examination; HAMD: Hamilton Depression Scale; HAMA: Hamilton Anxiety Scale; RBDQ-HK: REM sleep behavior disorder questionnaire-Hong Kong; ADL: Activity of Daily Living Scale; BMI: Body Mass Index; PINK1: PTEN induced putative kinase 1; P-TBK1: phosphorylated TANK binding kinase 1; PGAM5: phosphoglycerate mutase 5; BNIP3: BCL2 interacting protein 3. [a] Less participants took the examination of plasma p-TBK1 and BNIP3 (a-Syn+: HC = 11, PD = 46, PDs = 8; a-Syn-: HC = 20, PD = 19, PDs = 6). $^{*}P < 0.05$, $^{**}P < 0.01$, $^{***}P < 0.001$, plasma-derived biomarker levels compared to the negative a-Syn status with two sample T test.
